# Personalizing Drug Selection Using Advanced Clinical Decision Support

**DOI:** 10.4137/BII.S2506

**Published:** 2009-06-23

**Authors:** John Pestian, Malik Spencer, Pawel Matykiewicz, Kejian Zhang, Sander Vinks, Tracy Glauser

**Affiliations:** 1 Clinical Linguistic Group, Division of Biomedical Informatics.; 2 Division of Human Genetics.; 3 Division of Clinical Pharmacology.; 4 Division of Neurology. Department of Pediatrics, Cincinnati Children's Hospital Medical Center, University of Cincinnati, Cincinnati, OH 45229.

**Keywords:** clinical decision support, biomedical informatics

## Abstract

This article describes the process of developing an advanced pharmacogenetics clinical decision support at one of the United States’ leading pediatric academic medical centers. This system, called CHRISTINE, combines clinical and genetic data to identify the optimal drug therapy when treating patients with epilepsy or Attention Deficit Hyperactivity Disorder. In the discussion a description of clinical decision support systems is provided, along with an overview of neurocognitive computing and how it is applied in this setting.

## Background

Personalized medicine promises to improve the quality of patient care. That promise presumes that integrating genetic information into clinical decision support systems will provide physicians with new perspectives as they diagnose and treat patients. Guttmacher and colleagues note, “genomic-based knowledge and tools promise the ability to approach each patient as the biological individual he or she is, thereby radically changing our paradigms and improving efficacy. They go on to say, however, that we should expect only modest changes to result from genetics-based medicine, because ”personalized medicine has always been a component of good medical practice. Genetic tests may provide new tools, but they do not change the fundamental goal of clinicians to adapt available medical tests and technologies to the individual circumstance of their patients”.^1^

Nevertheless, there has been great progress in developing personalized tests. At the DNA level, 598 genetic labs currently test for 1,729 diseases. Of those tests, 1,449 are done for clinical care and 280 are done for research purposes (http://www.genetests.org).^2^ Whole-genome association studies for finding genetic predisposition markers for common, complex diseases are now being developed.^3,4^ Using these data, however, in a rapid-paced, clinical care setting requires innovation in their collection, integration and presentation. Our response to this challenge is to develop the Children's Hospital Resource In Selecting Therapy Individualized Expert (CHRISTINE).

CHRISTINE was developed at the Cincinnati Children's Hospital Medical Center (CCHMC), a leading pediatric academic medical center in Cincinnati, OH USA. CCHMC has approximately one million patient encounters annually and is one of the top three centers of pediatric research in the United States. CCHMC has a strong translational research culture that fosters such initiatives as CHRISTINE. Funding for CHRISTINE was provided from private donors and the State of Ohio's Third Frontier program. The original purpose of CHRISTINE was to support a clinician's decision with accurate and timely information related to drug selection for patients with Attention Deficit and Hyperactivity Disorder (ADHD) or epilepsy. An additional module for major mood depression has been commissioned. CHRISTINE includes four computational cores: an expert systems core for tracking expert opinion, a neurocognitve core for identifying the drugs, a data integration core and a user interface core. These are described below.

### The Electronic Medical Records

As early as the 1970's scientists began to see the value of capturing patient data in an electronic form.^5^ In 1988, McDonald formally conceptualized the impact that electronic data would have on patient care: “Three kinds of benefits may be expected: (1) improved logistics and organization of the medical record to speed care and improve care givers’ efficiency, (2) automatic computer review of the medical record to limit errors and control costs, and (3) systematic analysis of past clinical experience to guide future practices and policies”.^6^ As research continued, the value of electronically captured clinical data became evident. Eventually, Clinical Decision Support (CDS) systems began to emerge. Clinical decision support systems were described as having one or more of the following characteristics: (1) making patient data more apparent and accessible, (2) facilitating optimal problem solving and decision making, (3) providing support by presenting knowledge to physicians, nurses, laboratory technologists, pharmacists, patients, or other individuals in clinical practice, preventive care, or during training, (4) selecting or creating pertinent knowledge based on patient-specific data, (5) resulting in actions such as alerts or recommendations.^7^ This conceptual view helped form a framework for CDS system research and development.^8–13^ In this paper, however, we propose an alternate definition: A CDS should provide all relevant information in a way that supports and promotes accurate clinical decisions. *All relevant information* means only information that is germane to that particular decision. Ancillary information is excluded, but quickly and easily available. *Information* is not data; information is usable knowledge, whereas, data are its substrate. *In a way that supports* refers to a method that is easily understood by the decision maker. The method can be textual; graphic, like visual languages; or auditory, like warning sounds. CHRISTINE's approach to meeting this enhanced definition is described below.

### Neurocognitive Approach

**Figure 1:**
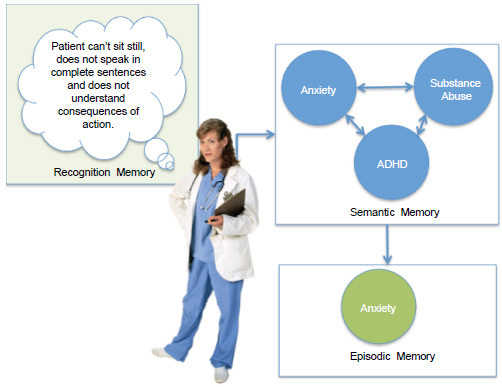
Neurocognitive process.

Our decision making process model involves recognition, semantic and episodic forms of memory.^14^ *Recognition memory* is the “judgment that a stimulus event has been previously experienced.^15^ As a meaning is represented through natural language, the relationships and features of that representation become known. For example, hearing that “a patient can't sit still, starts but rarely finishes things, and acts without thinking about the consequences” primes recognition memory to think about possible diagnoses and treatment. That priming and the subsequent recognition of symptoms orders multiple memories into a *semantic network*, whose nodes are linked together by relationships that have developed though experience. In this example, the physician may first think of ADHD but also consider such competing diagnoses as Generalized Anxiety Disorder and substance abuse withdrawal. The validity of these nodes is tested, for example by conversation with the patient's mother. An important aspect of semantic memory is that it can be used inferentially^16^ that is from general premises it can arrive at a necessary and specific conclusion. Next a conclusion or episode is selected. This final stage is called *episodic memory*. In this example, ADHD is selected as the specific episode because there are no signs of anxiety, (worry, stomach ache or headache) or substance abuse (weight loss, agitation, exhaustion). Now that a decision has been made, treatment must be considered. Traditionally, the next step would be to think of the various pharmacogenetic drug choices. The semantic network for this decision may include the patient's age, weight and gender, other medications being taken and the insurance formulary. Titration will depend on a number of characteristics, including the patient's ability to metabolize the drug. This process is depicted in Figure 1.

Specific sections of the patient's genetic structure affect how well the patient metabolizes a particular drug. Many of the enzymes involved in drug metabolism belong to the large group known as cytochrome P450 isoenzymes (abbreviated CYP), which are involved in metabolism of a number of drug classes. This very large group has evolved many genetic variants in human populations. For example, isoenzymes encoded by CYP2D6 and CYP2C19 genes are responsible for the metabolism of many commonly prescribed medications (e.g. antipsychotics, antidepressants). Certain variants are ultra-metabolizers and other variants are low or poor metabolizers. Should the original drug provide most efficacy then ultra-metabolizers may require higher dosages since they metabolize specific drugs much faster than low-metabolizers would. Low metabolizers, on the other hand, may require lower dosages since they metabolize the drugs more slowly.^17,18^ Our Human Genetics Laboratory has applied these consideration with over 5,000 patients, and much of the logic is or will be incorporated into CHRISTINE.

**Figure 2:**
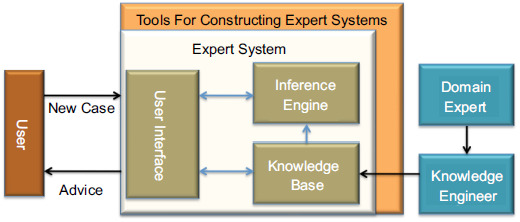
CHRISTINE expert system framework.

In the ADHD example, one clinician makes the final diagnostic decision based on her or his knowledge and experience. Then a treatment decision is made. With a neurocognitive approach it is possible to combine the knowledge of multiple experts into a centralized database or expert opinion module, and then use that combined knowledge to support decisions about diagnoses or treatments. CHRISTINE can cover genetic, clinical and environmental information in the expert opinion module and model it according to decision criteria–-criteria that quickly become complex. Figure 2 depicts the conceptual framework of this approach. On the right of the figures, domain experts provide information to knowledge engineers, who curate the information so it can be entered into the knowledge base. The inference engine then waits for a call by the user interface. The data are searched and any relationships are found. The results flow back to the user.

Using this approach has many benefits: permanence, reproducibility, efficiency, and consistency. In the context of medicine, Coiera summarizes the benefits of such a system: (1) improved patient safety, (2) improved quality of care, and (3) improved efficiency in health care delivery.^19^

Efficiency in decision-making is important–-ideally modeling an expert's decision making process with the least amount of relevant information. For example, Modeling an expert's decision process about a disease for which the decision considers two factors, e.g. age group and gender, if the model has 6 age groups and 2 genders there will be 12 scenarios. If there are 10 useful therapies for this disease and a single rating factor, then 120 data points would be required to emulate an expert's selection of the therapy. Those datapoints, however, are useless for understanding the reasoning behind the selection. A report stating that the selection of Concerta was based on the patient being an adolescent female (the patient's specific “condition”) is not as useful as a report stating that Concerta was selected because of its utility as measured by its ranking against: drug practical issues, drug effect on patient co-morbidities, and drug-related idiosyncratic reaction.

Rather than rating conditions directly, the system rates conditions in the context of clinical criteria. For example, two important considerations in selecting a therapy are the drug's effect on a patient's cardiovascular function and the drug's effect on a patient's endocrine function. In the case of epilepsy, for which both of those considerations are based primarily on age group, that factor is rated in two different contexts. We refer to these contextual considerations as categories. For the above example, in a system with 50 categories, the worst-case data point calculation becomes: 50 categories × 6 age groups × 2 genders × 10 useful therapies = 6000 data points. Attempting to describe a selection process involving 50 categories would be difficult, so categories are grouped into classes. It is the description of each class that is presented as the criterion the for therapy selection. Here is where CHRISTINE provides support. In fact, one important goal of CHRISTINE is to extend such information in a usable format to community pediatricians who do not have this level of expertise available.

## Development Methods

There are a number of methods for software development: iterative, agile, extreme programming, and waterfall. Each has a particular role that is contingent upon the size and complexity of the project and the organizational setting. We used the *agile* method. Its basic approach is iterative: it incrementally delivers increasing portions of a software project, one step at a time.^20^ Agile development methods promote development iterations, teamwork, collaboration and process adaptability throughout the life cycle. All of them, however, follow the same pattern: requirements gathering, specifications, architecture, design, implementation and testing, deployment and maintenance. The agile method employs steps that are completed in “time-boxes.” Teamwork is vital to this method. The team's size and membership can either increase or reduce the chance of success. Too large a consensus is a challenge; too small, and the required expertise leaves the development vacuous. Ideally, a team is made of five to nine members, with one domain expert representative.^21^ In our case, using a single domain expert would have eliminated the multi-domain expertise needed to capture specialized medical knowledge. Hence CHRISTINE drew from international experts in human genetics, pharmacogenetics, epilepsy, ADHD, nursing, computer science, machine learning and text mining. Introducing this level of expertise also introduced rigorous debate. In the end, many more iterations were needed for requirement gathering and software development, iteration that we believe improved the quality of the overall project.

### Architecture

At CHRISTINE's core is a decision-support, expert system. The goal of the expert system is to “emulate the search behavior of human experts in solving a problem”.^16^

A common implementation strategy for expert systems is to separate the delivery of knowledge from the knowledge itself. That is useful for several reasons. First, knowledge engineers and software developers can implement the system in parallel. Deployment and ongoing maintenance can occur in parallel, as well. Second, the knowledge can be decoupled from a particular implementation language or framework, thus making it portable, interoperable, and resilient to change. Software developed without this decoupling is often referred to as *conventional software*. Conceptual differences between conventional software and expert systems are detailed in Table 1.^22^

**Table 1:** Comparison of programming functions.

Characteristic	Conventional program	Expert system
Control by …	Statement order	Inference engine
Control and data	Implicit integration	Explicit separation
Control strength	Strong	Weak
Solution by	Algorithm	Rule and inference
Solution search	Small or none	Large
Problem solving	Algorithm is correct.	Rules
Input	Assumed correct	Incomplete, incorrect
Unexpected input	Difficult to deal with	Very responsive
Output	Always correct	Varies with problem
Explanation	None	Usually
Applications	Numeric, file and text	Symbolic reasoning
Execution	Generally sequential	Opportunistic rules
Program design	Structured	Little or no structure

For CHRISTINE, an expert system was necessary for several reasons: the data are often fuzzy and lack solid structure; there is a need for symbolic reasoning; rules and inferences exist but will change with new medical knowledge; the general knowledge available for decision support is large; unexpected inputs should be anticipated and accepted. Because medical knowledge continues to change, one should expect the CHRISTINE system to be modified.

Expert systems of production quality usually offer features that address barriers to end-user adoption. The first such feature is an explanation system: software designed to convey the reasoning behind the recommendations that an expert system offers. Second is a way to get new knowledge into the system or to make corrections to existing knowledge. Finally, use of the system should fit comfortably within the workflow of the clinician. Although expert systems have widespread use across many disciplines, relatively few expert system shells are available that are production quality and are web-based, free or open source.

### Requirements Gathering

The CHRISTINE system and its underlying software architecture have evolved steadily because of our adherence to a strict set of design requirements. These requirements and the cores where they are most important are listed in Table 2.

**Table 2:** Requirements.

Requirement	Core	Explanation
Simplicity	All	Simple systems are easier to implement, document, deploy, and maintain. Less code is easier to review, explain and refactor.
Design for testing	Expert and data integration	The system should be designed so that all features are easily accessible by automated testing software. The proper working of the software should be easily verifiable.
Reusable components	Expert and user interface	To simplify development, the system utilizes a layered and plug-in architecture. At the lowest level, core libraries provide important basic functionality. An application framework rests on the core libraries and is designed to receive and respond to service requests. Processing is handled via service-specific plug-ins. The core libraries are partitioned into features. Each feature is stored in its own directory tree. Using an innovative code weaving system, our installation tool is able to select which features are to be integrated.
Separation of form and function expert and data integration	User interface	The visual design is as important as the software design. To provide the flexibility necessary to create an optimal visual presentation, we make extensive use of templates and CSS technology.
Adaptable to changes in medical knowledge	Expert and data integration	Medical knowledge grows at a rapid pace. Some of this knowledge must be incorporated in CHRISTINE.
Open source	All	CHRISTINE should be developed using open source software.

### Work Flow

**Figure 3:**
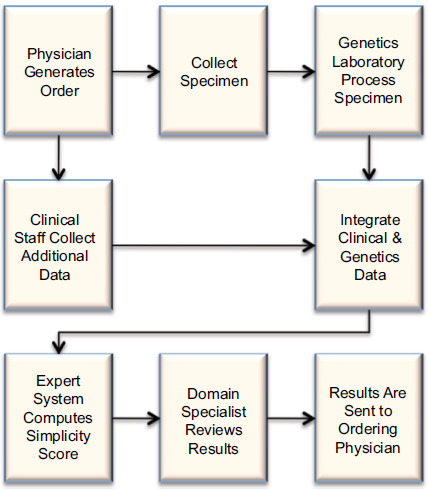
CHRISTINE workflow.

How does a workflow using advanced decision support differ from the example of a clinical case described earlier? Well, the physician, the patient, and the patient's parent meet. The physician still must decide the best diagnosis and therapy. The workflow diverges, however, in how the data are managed and analyzed. It is here that computational resources can be used to support the decision. Figure 3 provides a graphical representation of this workflow. On the left side is the data integration core. On the right side is the expert core. In the data integration section, a physician's order is generated and a specimen is sent to the Molecular Genetics Laboratory, which uses it to identify a panel of single nucleotide polymorphisms, which predict a patient's metabolism rate of a certain medication. The results of this analysis are reviewed and then sent to CHRISTINE's centralized database. Simultaneously with this activity, the physician or other clinical staff completes a series of questions about the patient. These data are then integrated into CHRISTINE's centralized database. Upon completion of the data gathering, the neurocognitive expert conducts a series of computations. The results are provided to a domain specialist who conducts a final review before communication with the patient's physician. Finally, the specialist communicates the results to the physician by sending a report like the one found in Figure 5.

### Specifications and Development

Specifications for the software are found in Table 3. This table shows the factors considered, descriptions of the tools used and some strengths and weaknesses of these tools. As explained, an overarching requirement was the use of open source software. This requirement was violated only once, in choosing the PDF report generator. After experimentation with PHP PDF generators, html2pdf and dompdf, we chose the proprietary system PD4ML for its stability and reliability. This was the only choice that did not use open-source software.

**Table 3:** Specifications.

Factor	Description	Strength	Weakness
Database	MySql	Open source, stable, large user base, well suited for web applications, fast, scalability	Missing some SQL features
Language	Perl	Open source, unix functionality, strong report generation, full featured scripting, regular expressions, rapid prototyping, many ways to do something	Steep learning curve, many ways to a solution
Revision control	Subversion	Open source, distributed development, CVS de-facto replacement, versioned directories, atomic commits, merge tracking, well developed security	Distributed approach may lose tight management control, no network bottleneck management
Web application error testing	Selinium	Open source, designed for web applications, works in browser-like users, works on multiple platforms, record and playback	No object mapping, no object identity tool, no database tests
Webserver	Apache	Open source, large user group, secure, stable and reliable	Large code base, potentially slow
Operating system	OSX	Built on BSD, highly compatible with Linux, large user base, integrated tool set (apache, perl), extensive support, stable	Not open source, precompiled binaries not available or lag in version
PDF licensing	PD4ML	Low cost, but not open source	Not open source Complicated

Total project development occurred over approximately 24 months. The actual coding took approximately 12 months, using 1.5 FTE. The remaining time was for administrative overhead. CHRISTINE has a centralized user interface that includes visual language components, which provide examples of how color is used as a visual language.

There are four distinct roles necessary to complete a pharmacogenetic test in CHRISTINE. These roles are: *clinical*–-the clinician who enters the clinical information, *lab*–-the laboratory personnel who conduct the test, *signoff*–-the person responsible for verifying the accuracy of the results, and the *finisher*–-the physician responsible for authorizing final approval and release of the information.

### Inputs and Outputs

**Figure 4:**
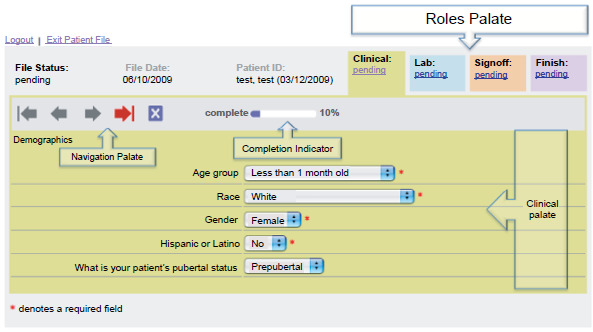
CHRISTINE graphical user interface.

Figure 4 shows a typical user interface for CHRISTINE. The master palate has multiple, context-sensitive palates. In this case there are navigation, clinical and roles palates. The palates adjust to the users’ roles.

**Figure 5:**
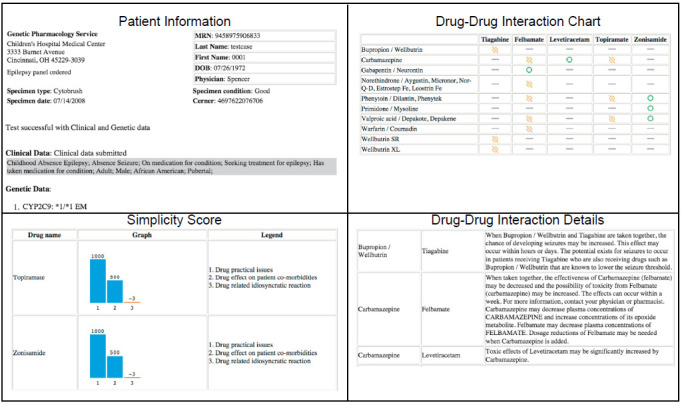
Report examples.

The lighter hues signify that an incomplete activity exists. The darker hues show completed activity. The hues are also attached to the words pending or completed. This visual approach is also seen in the final reports. In Figure 5 the importance of each category is represented with a bar chart. Drug-drug interaction is represented with universal symbols. Readers can go to http://ncc.cchmc.org/christine to see the full system.

CHRISTINE produces a number of informative and exhaustive reports. The reports are organized with the graphical views in the beginning and details about the views presented afterwards. They include patient information, genetic information, drug options listed by simplicity score, graphical depiction of drug-drug interaction and drug-drug interaction details. A collation of these reports is found in Figure 5. The upper left corner shows summary patient information. The lower left corner shows two drug selections and their related simplicity indices. The upper right corner provides information about drug-drug interaction. In this report a circle with a line through it indicates a negative outcome, a green circle indicates no known problem, and a dash indicates there is no interaction information available. The lower right corner provides detailed information about those interactions.

## Testing and Implementation

Our infrastructure meets a very high level of stability. All systems run from our 15,000 square-feet data center that consistently maintains 99.999% reliability. So, there was no intention of testing infrastructure stability or reliability. Rather, the main focus was on validity testing of CHRISTINE's results. The first stage examined whether CHRISTINE's genetic algorithms yielded the same results as those from the Genetics laboratory, which had tested these algorithms on over 5,000 pediatric patients. Data from known results (n = 40) were entered into CHRISTINE. The results showed that CHRISTINE's algorithms matched the Genetics lab output 100%. The next step was to determine whether CHRISTINE's overall algorithms matched expert opinion. Again a series of known patient cases (n= 10) were entered into CHRISTINE. An expert was then asked to decide on the drug selection, using the same data. The expert and CHRISTINE matched 100% of the time. The next step of the implementation was to test the user interface on end users. Although we had iterated that process three times in its development, this final iteration was appropriate. The end users, in our case two pediatric practices, had minor requests, which included making the report easier to understand in part by including graphic representation of a drug's factors and also a drug-drug interaction report, as shown in Figure 5.

## Discussion and Lessons Learned

### Complexity

Clinicians, not machines, are responsible for clinical decisions. Advanced clinical decision support systems like CHRISTINE are designed to support those decisions. This is no small task. The simple example offered herein has shown that with, as little as, two factors, over 120 decision points must be considered. In the not too distant future maintaining these data will be challenging enough to require a semi-automated method for scanning scientific literature and selecting those factors that are germane to the decision. We call this tool an *artificial expert.*

### Time Commitment

Completing the expert module questions is labor intensive. Full completion required about 20 hours. In our case we relied on the goodwill of colleagues. This method, however noble, extended the timeline to what would be unacceptable in a production environment. Future efforts should include some form of incentive.

### Modular Approach

It is important to construct a modular system as a framework. The rapid change in emerging clinical knowledge will require regular updates to length, width, and depth of the knowledge base. Using this approach we have been able to add clinical modules, adolescent suicide linguistic analysis and other well-known analytical tools. Had we not developed this framework in advance, CHRISTINE would be a stand-alone system.

### Proven Value

CHRISTINE has proven itself technically. The remaining unanswered questions about its influences, if any, on physician behavior. Will it enable better patient outcomes because it is intended to provide information for personalized care? Will it improve the economics of care? These and related questions call for formalized research by health services researchers.

### Team Management

The most challenging task for the project manager was to manage the team. Teams of this nature are highly creative and eager to discuss their ideas. That is a valuable asset in developing advanced technology; it also, however, spawns a tendency for scope creep. Overall project management should be the responsibility of a respected clinician who can continually keep the project of track.

## Disclosure

The authors report no conflicts of interest.

## References

[1] Guttmacher A.E., Porteous M.E., Mclnerney J.D. Educating health-care professionals about genetics and genomics. Nat Rev Genet. 2007; 8(2): 151–7. 17230201 10.1038/nrg2007

[2] Ginsburg G.S., McCarthy J.J. Personalized medicine: revolutionizing drug discovery and patient care. Trends Biotechnol. 2001; 19(12): 491–6. 11711191 10.1016/s0167-7799(01)01814-5

[3] Cargill M., Altshuler D., Ireland J. Characterization of single-nucleotide polymorphisms in coding regions of human genes. Nat Genet. 1999; 22(3): 231–8. 10391209 10.1038/10290

[4] Halushka M.K., Fan J.B., Bentley K. Patterns of single-nucleotide polymorphisms in candidate genes for blood-pressure homeostasis. Nat Genet. 1999; 22(3): 239–47. 10391210 10.1038/10297

[5] Jenkin M.A., Cheezum L., Essick V. Clinical patient management and the integrated health information system. Med lnstrum. 1978; 12(4): 217–21. 357938

[6] McDonald C.J., Tierney W.M. Computer-stored medical records. Their future role in medical practice. JAMA. 1988; 259(23): 3433–40. 3286915

[7] Greenes R. Clinical Decision Support: The road ahead. Burlington, MA: Elsevier; 2007.

[8] Eibling D. Making us smart: why the design of clinical decision support systems is so critical. Laryngoscope. 2008; 118(12): 2121–4. 19029849 10.1097/MLG.0b013e31818ee143

[9] German E., Leibowitz A., Shahar Y. An architecture for linking medical decision-support applications to clinical databases and its evaluation. J Biomed Inform. 2008. Nov 7. 10.1016/j.jbi.2008.10.007 19027088

[10] Harrison J.P., McDowell G.M. The role of laboratory information systems in healthcare quality improvement. Int J Health Care Qual Assur. 2008; 21(7): 679–91. 19055276 10.1108/09526860810910159

[11] Lipton J., Hazelzet J.A. Clinical decision support systems: Important tools when appropriately used. Pediatr Crit Care Med. 2009; 10(1): 128–9. 19131870 10.1097/PCC.0b013e31819838f9

[12] Mack E.H., Wheeler D.S., Embi P.J. Clinical decision support systems in the pediatric intensive care unit. Pediatr Crit Care Med. 2009; 10(1): 23–8. 19057443 10.1097/PCC.0b013e3181936b23

[13] Taffel B. From data interoperability to value-driven healthcare. Tenn Med. 2008; 101(12): 31. 19097352

[14] Duch W., Matykiewicz P., Pestian J. Neurolinguistic approach to natural language processing with applications to medical text analysis. Neural Netw. 2008; 21(10): 1500–10. 18614334 10.1016/j.neunet.2008.05.008 PMC2633093

[15] Rugg M.D., Curran T. Event-related potentials and recognition memory. Trends Cogn Sci. 2007; 11(6): 251–7. 17481940 10.1016/j.tics.2007.04.004

[16] Quinlan R.M. Semantic Memory. Cambridge: Bolt Beranek and Newman; 1966.

[17] Kirchheiner J., Nickchen K., Bauer M. Pharmacogenetics of antidepressants and antipsychotics: the contribution of allelic variations to the phenotype of drug response. Mol Psychiatry. 2004; 9(5): 442–73. 15037866 10.1038/sj.mp.4001494

[18] Roots I., Gerloff T., Meisel C. Pharmacogenetics-based new therapeutic concepts. DrugMetab Rev. 2004; 36(3-4): 617–38. 10.1081/dmr-200033458 15554239

[19] Coiera E. Guide to Health Informatics. 2 ed: A Hodder Arnold Publication; 2003.

[20] Larman C., Basili V. Iterative and Incremental Development: A Brief History. Computer. 2003; 36(6): 47–56.

[21] Boehm B., Turner R. Balancing Agility and Discipline: A Guide for the Perplexed. Addison-Wesley; 2004.

[22] Giarratano J., Riley G. Expert Systems: Principles and Programming, Fourth Edition: Course Technology; 2004.

